# Anesthetic considerations for blue rubber bleb nevus syndrome: a case report

**DOI:** 10.1186/s40981-019-0304-4

**Published:** 2019-12-16

**Authors:** Mariko Aizawa, Satoshi Ishihara, Takeshi Yokoyama

**Affiliations:** Department of Anesthesia, Teine Keijinkai Hospital, 1-12-1-40 Maeda, Teine, Sapporo, 006-8555 Japan

To the Editor,

Blue rubber bleb nevus syndrome (BRBNS) is a rare condition characterized by numerous malformations of the venous system in the skin and visceral organs [1]. Surgical resection of the gastrointestinal tract is the choice of treatment for bleeding from venous malformations [2], and it requires general anesthesia. In general, anesthetic management in patients with BRBNS is challenging because of vascular malformations, especially in the airway and spinal canal [3–5]. Here, we report the case of a patient with BRBNS who underwent open abdominal surgery with successful general and epidural anesthesia.

A 60-year-old woman with BRBNS was scheduled for curative intestinal resection via laparotomy for bleeding hemangiomas in the ileum. In addition to routine history taking and physical examinations, preoperative evaluation was focused on two main areas. First, the extent of airway involvement was evaluated. Multiple hemangiomas were found on physical examination (Fig. 1), but no previous airway bleeding or obstruction was noted. The airway was assessed using a fiber optic bronchoscope, which revealed multiple hemangiomas in the larynx and trachea (Fig. 2). Tracheal stenosis or bleeding was not noted. Second, the feasibility of epidural anesthesia was evaluated. There was no particular history or physical findings suggesting a spinal canal lesion. Additionally, we assessed the spinal canal including epidural space using magnetic resonance imaging (MRI), which revealed no vascular lesion. Typical venous hemangiomas were found in the subcutaneous tissue and muscles of the back. An epidural catheter was inserted at the T10–T11 interspace using a 17-gauge Tuohy needle after confirming the absence of lesions in the soft tissue on ultrasonography. General anesthesia was then induced. Mask ventilation was performed without any issue. Laryngoscopy and tracheal intubation were uneventfully performed using a McGRATH MAC video laryngoscope with a size 3 blade (Aircraft Medical, Edinburgh, UK), to assist in minimizing mechanical contact with the hemangiomas in the upper airway. Upon intubation, using a fiber optic bronchoscope, we confirmed that the tracheal tube was not in contact with any vascular lesions of the trachea and that there was no bleeding. The surgery was completed uneventfully. The patient was extubated without disruption of any upper airway lesions. The postoperative course was uneventful.

**Fig. 1:**
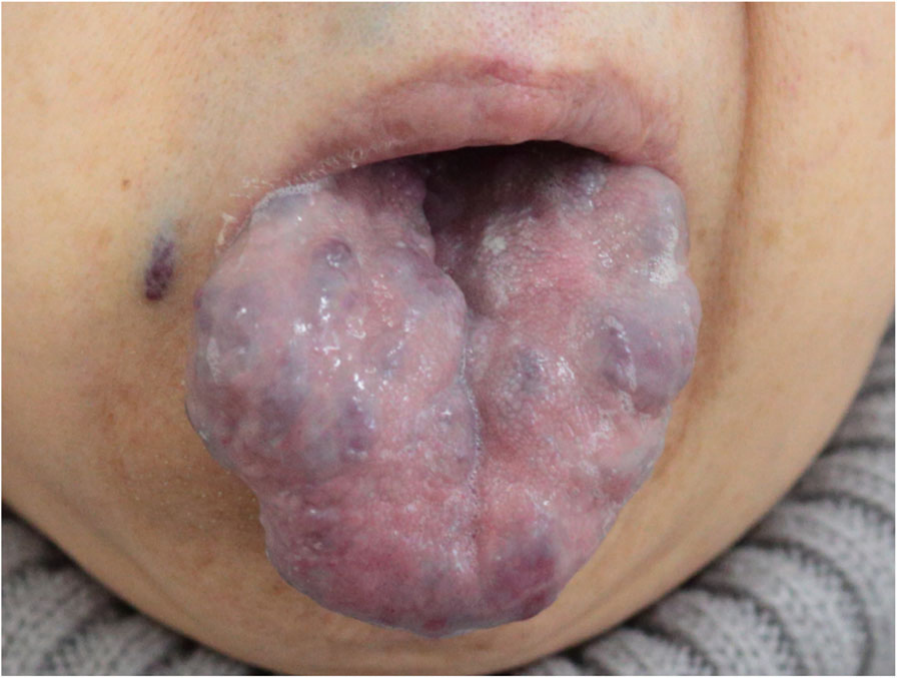
Multiple hemangiomas on the tongue

**Fig. 2:**
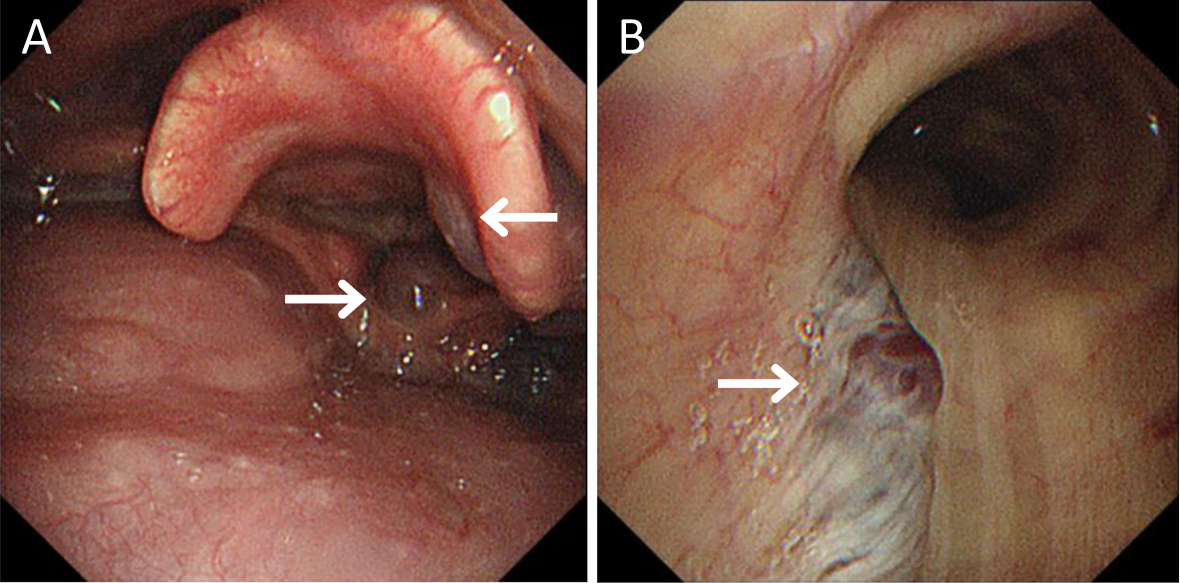
Fiber optic bronchoscope showing multiple hemangiomas (**a**) at the epiglottic vallecula and arytenoid (white arrows) and (**b**) in the trachea (white arrow)

The present case illustrates the importance of careful preoperative evaluation of the upper airway and spinal canal in patients with BRBNS. Airway management can be problematic at general anesthesia because airway hemangiomas can cause airway obstruction or bleeding [6]. As for spinal canal lesions, epidural anesthesia might cause bleeding and hematoma formation if they are overlooked.

With regard to airway evaluation, focused history taking and physical examination are essential, and use of a fiber optic bronchoscope is reasonable. Oral intubation is feasible, and video laryngoscope is useful for safe intubation [4, 5, 7]. Epidural anesthesia can be safely managed by paying attention to the possibilities of spinal canal lesions. MRI is the preferred modality for evaluating spinal canal involvement [8]. Although challenging, anesthesia can be safely performed in patients with BRBNS. Thorough preoperative evaluation based on the understanding of the nature of BRBNS is essential.

## Data Availability

The data that support the findings of this study are available on request from the corresponding author MA.

## Abbreviations

BRBNS: Blue rubber bleb nevus syndrome
MRI: Magnetic resonance imaging
